# Psychological and physiological stress and burnout among maternity providers in a rural county in Kenya: individual and situational predictors

**DOI:** 10.1186/s12889-021-10453-0

**Published:** 2021-03-06

**Authors:** Patience A. Afulani, Linnet Ongeri, Joyceline Kinyua, Marleen Temmerman, Wendy Berry Mendes, Sandra J. Weiss

**Affiliations:** 1 Epidemiology and Biostatistics Department, University of California, San Francisco (UCSF), 550 16th St, 3rd Floor, San Francisco, CA 94158 USA; 2 UCSF Institute for Global Health Sciences, San Francisco, USA; 3 Kenya Medical Research Institute, Nairobi, Kenya; 4 The Aga Khan University Medical College, Nairobi, Kenya; 5 UCSF Department of Psychiatry, San Francisco, USA; 6 UCSF Department of Community Health Systems, San Francisco, USA

**Keywords:** Stress, Burnout, Heat rate variability, Cortisol, Maternity providers; Kenya

## Abstract

**Background:**

Stress and burnout among healthcare workers has been recognized as a global crisis needing urgent attention. Yet few studies have examined stress and burnout among healthcare providers in sub-Saharan Africa, and even fewer among maternity providers who work under very stressful conditions. To address these gaps, we examined self-reported stress and burnout levels as well as stress-related physiologic measures of these providers, along with their potential predictors.

**Methods:**

Participants included 101 maternity providers (62 nurses/midwives, 16 clinical officers/doctors, and 23 support staff) in western Kenya. Respondents completed Cohen’s Perceived Stress Scale, the Shirom-Melamed Burnout scale, and other sociodemographic, health, and work-related items. We also collected data on heart rate variability (HRV) and hair cortisol levels to assess stress-related physiologic responses to acute and chronic stress respectively. Multilevel linear regression models were computed to examine individual and work-related factors associated with stress, burnout, HRV, and cortisol level.

**Results:**

85% of providers reported moderate stress and 11.5% high stress. 65% experienced low burnout and 19.6% high burnout. Average HRV (measured as the root mean square of differences in intervals between successive heart beats: RMSSD) was 60.5 (SD = 33.0) and mean cortisol was mean cortisol was 44.2 pg/mg (SD = 60.88). Greater satisfaction with life accomplishments was associated with reduced stress (β = − 2.83; CI = -5.47; − 0.18), while motivation to work excessively (over commitment) was associated with both increased stress (β = 0.61 CI: 0.19, 1.03) and burnout (β = 2.05, CI = 0.91, 3.19). Female providers had higher burnout scores compared to male providers. Support staff had higher HRV than other providers and providers under 30 years of age had higher HRV than those 30 and above. Although no association between cortisol and any predictor was statistically significant, the direction of associations was consistent with those found for stress and burnout.

**Conclusions:**

Most providers experienced moderate to high levels of stress and burnout. Individuals who were more driven to work excessively were particularly at risk for higher stress and burnout. Higher HRV of support staff and providers under age 30 suggest their more adaptive autonomic nervous system response to stress. Given its impact on provider wellbeing and quality of care, interventions to help providers manage stress are critical.

**Supplementary Information:**

The online version contains supplementary material available at 10.1186/s12889-021-10453-0.

## Background

Stress and burnout among healthcare workers have been recognized as global crises that need urgent attention [1, 2]. Stress involves psychological and physiological responses to environmental stressors—the causes of stress—over which people often have no control [3]. Perceived stress affects both the autonomic nervous system (ANS) and the hypothalamic-pituitary-adrenal (HPA) axis – two major stress response systems that are tasked with managing the body’s physiologic response to stress [4, 5]. How people perceive stressors is particularly important to the physiological response, as people exposed to the same stressors may perceive them differently, resulting in a different physiological response [6].

Prolonged stress without adequate coping mechanisms leads to burnout, which is characterized by both physical and emotional exhaustion [7, 8]. Healthcare workers are particularly prone to burnout due to the emotionally demanding nature of their work. Burnout among healthcare workers also manifests as depersonalization (feelings of negativism, cynicism, or detachment from one’s job), feelings of helplessness, and reduced professional efficacy [9]. Burnout affects interpersonal skills, job performance, and psychological and physical health. Prolonged high stress and burnout, therefore, can lead to lower productivity and effectiveness, decreased job satisfaction, and reduced commitment to the job [10, 11]. These factors, in turn, result in poor quality of care, risks to patient safety, and poor attitudes towards patients [12–14]. Burnout among healthcare providers is associated with increased self-reported errors, reduction in time devoted to providing clinical care, and higher mortality rates for their patients [12, 15]. In addition, it leads to absenteeism and high staff turnover, which is expensive for the health care system [10, 12, 16].

High stress and burnout are also associated with poor health outcomes for the people experiencing it. These include psychiatric conditions such as depression [17, 18], anxiety [19], substance abuse [18, 20], and suicidality [21, 22], as well as cardiovascular disease, digestive disorders, poor quality of life, and premature mortality [1–4]. Thus, high stress and burnout are critical medical and public health problems, with profound consequences on individual providers as well as on the healthcare system [10, 23].

Recent research has highlighted that stress and burnout of maternity providers are key drivers in the disrespect and abuse of women during childbirth—which has also been recognized as a global crisis [24, 25]. Yet, few studies have assessed stress and burnout among maternity providers in Sub-Saharan Africa (SSA) and even fewer have done this in Kenya. A recent systematic review on burnout among health care workers in SSA reported high levels of burnout among nurses and doctors [26]. Only two studies focused on maternity providers (midwives, in these cases), and these were in Uganda [27] and Senegal [28]. Both reported high levels of burnout (over 50%) among midwives using different measures of burnout. A more recent study among nurses working in a large maternity hospital in Kenya also found 88.6% of the nurses were experiencing burnout measured with the Maslach Burnout Inventory-Human Services Survey (MBI-HSS) [29]. We are not aware of any studies examining physiological measures of stress among healthcare workers in Kenya.

Providers in Sub-Saharan Africa work under very stressful conditions [30–32]. For maternity providers, in particular, work-related stressors are numerous, including: an overwhelming work load from staff shortages; not being able to provide best practice due to lack of drugs, supplies, and equipment; being required to manage complications beyond their competency due to inadequate skill and a poor referral system; feelings of inadequacy in the face of high maternal and newborn mortality; financial strain from poor remuneration; poor working conditions with insufficient basic resources such as scarcity of water and sanitation; and disrespectful behavior from patients, colleagues, and superiors [30, 33, 34]. These situational factors related to job demands and resources have the potential to increase providers’ perceived stress, with implications for their work-related burnout and physiologic responses. However, few studies have examined how these factors may be associated with individual providers’ experiences of stress or burnout [27–29].

**Fig. 1:**
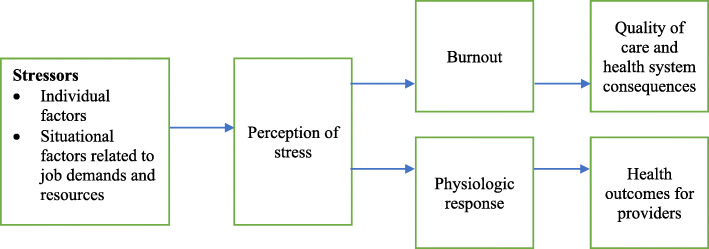
Conceptual model

We sought to address the dearth of research on stress and burnout among maternity providers in SSA. Our aims were to: (1) assess levels of stress and burnout among maternity providers and support staff in Kenya; and (2) identify individual and situational factors associated with provider stress, burnout and stress-related physiologic measures. We hypothesized that perceived stress, burnout and physiological measures are influenced by individual level factors (including demographic and socioeconomic factors) as well as by situational factors related to job demands and resources [35]. We also hypothesized that perceived stress mediates the relationship between these potential individual and situational stressors and burnout and physiological responses (conceptual model in Fig. 1). Our hypotheses were exploratory due to the lack of similar prior research in the setting on which to build. Although consequences and outcomes were not examined in this project, they are shown in the model to highlight potential implications of the aims we examined.

## Methods

Data are from a mixed-methods study with maternity providers in a rural county in western Kenya. The county is described in detail elsewhere [36]. It has eight sub-counties, each of which has a sub-county hospital, in addition to several health centers. The county population is about one million, with an estimated 40,000 births annually [37]. The county has a low healthcare worker to patient ratio, which is likely a key factor for stress and burnout. The provider/patient ratio in the county is 32 nurses, 19 clinical officers (non-physician clinicians trained to perform certain duties that usually require a medical doctor), and four doctors per 100,000 people respectively [38]. We use the term maternity providers in this study to refer to both clinical staff such as nurses, midwives, doctors, and clinical officers as well support staff, including nurse aides and cleaners working in maternity units (providing antenatal, intrapartum, and postnatal care and support services). We included support staff because they have been shown to play an important role in maternity care and women’s experiences in other studies [39, 40].

### Procedures

The primary data used in the current analysis were collected through a survey—structured individual interviews with 101 maternity providers—along with physiologic assessments of the ANS using a measure of heart rate variability (HRV) and the HPA axis through measurement of hair cortisol. Data was collected between June and September 2019. We purposively recruited providers from 30 health facilities in the county representing those facilities with the highest volume of births: the county hospital, all the sub-county hospitals, and two to three other facilities in each of the eight sub-counties. The goal in each facility was to recruit a minimum of one or two clinical officers (if the facility had any), two or more nurses depending on the number of nurses available, and one or two support staff (nurse aids and cleaners). Two female Kenyan research assistants (RAs) with bachelor’s degrees collected all the data.

The study was approved by the Institutional Review Boards for Protection of Human Subjects of University of California, San Francisco and Kenya Medical Research Institute, and by the Kenya National Commission for Science, Technology & Innovation (NACOSTI). Approval for the study within Migori was granted by the County Commissioner and the County Director of Health. Informed consent was obtained from all participants for each study component (i.e., interview, heart rate variability, hair sample).

Interviews were conducted in English, Swahili, and Dholuo to acquire data on stress and burnout as well as potential individual and situational stressors. All data collection tools were translated and piloted with potential respondents prior to the actual study. Between three and five providers participated in the interviews in most facilities and they lasted about 40 to 60 min. Response rate for the interview was 100%. Heart Rate Variability was measured for all respondents using the CorSense monitor by Elite HRV^1^ in the middle of the interview—immediately after they responded to a series of questions regarding stress and burnout and before the individual and situational predictors. The sensor was placed on the respondent’s index finger, with readings transmitted via Blue tooth to the Elite HRV app on the tablet. The reading was taken in the seated position for 5 min, which is considered acceptable for short-term HRV readings [41, 42]. After participants completed the interview, the RA asked for permission to take a sample of their hair from the scalp at the back of the head. The sample consisted of approximately 40–50 strands, 3–4 mm thick, and 3 cm long; it was wrapped in aluminum foil and stored in a ziplock bag for later cortisol assay. In addition to these individual provider measures, a facility-level survey was also administered to the head of the maternity unit to obtain data on facility-level indicators.

### Measures

#### Dependent variables

Our psychological measures included two self-reported questionnaires assessing perceived stress and burnout. Measures of physiologic stress comprised an assessment of two primary stress response systems: the provider’s current ANS state (heart rate variability) and an assessment of the provider’s longer term, HPA axis response in the months preceding data collection (hair cortisol).

##### Perceived stress

We measured perceived stress using the Cohen Perceived Stress Scale (PSS)—a global measure of perceived stress that has been validated in several countries [43], including in sub-Saharan Africa [44–46]. It has 10 items asking about feelings and thoughts during the last month to assess the degree of unpredictability, uncontrollability, and overload respondents experience in their lives (Additional file 1). The PSS has undergone substantial testing for its validity and reliability. Internal consistency of the PSS has ranged from alphas of 0.69–0.91 across global studies [43, 47]. A comprehensive psychometric analysis of the measure with an Ethiopian population indicated good factorial validity for the 10 item PSS as well as internal consistency, item discrimination, and convergent validity [48]. Validity and reliability have also been supported in Kenyan populations [46, 49]. The Cronbach alpha for our sample was 0.6. The summative PSS score ranges from 0 to 40, with scores of 0–13 considered low stress, 14–26 considered moderate stress, and 27–40 considered high perceived stress [43].

##### Burnout

The Shirom-Melamed Burnout Measure (SMBM) was also used [50]. This measure assesses the degree of emotional, physical, and mental exhaustion caused by stress. We used the 14 item version which had three subscales for physical fatigue (6 items), emotional exhaustion (3 items) and cognitive weariness (5 items) (Additional file 1). A mean burnout index is calculated for each participant, with scores ranging from 1 to 7 [50, 51]. The SMBM has undergone psychometric testing in various populations with strong evidence for its validity and reliability in different populations [50–53]. Reliability coefficients have exceeded 0.70 in most studies with adult workers in human service professions [50, 53]. Internal reliability testing with the sample in our study found a Cronbach alpha of 0.87. There are no specific cut offs for burnout. But a commonly used cut-off value for high or clinical burnout is ≥3.75, and ≤ 2.0 as no burnout [54, 55]. We thus considered ≤2.0 as no burnout, 2.1–3.74 as moderate burnout and ≥ 3.75 as high burnout. Scores for each subscale can be used as well.

##### Heart rate variability

HRV is a measure of the variation in beat-to-beat interval between consecutive heart beats. Our HRV assessment specifically acquired an estimate of cardiac vagal control [56, 57]. We evaluated the electrocardiogram (ECG) of each participant to determine the time between R waves (R-R interval) in the QRS complex. We used time domain measures because of their utility and simplicity in short-term assessments including: RMSSD (root mean square of successive differences in RR intervals), lnRMSSD (the natural log of the RMSSD) and SDNN (standard deviation of all normal RR intervals). These indices reflect parasympathetic activity of the ANS, with higher values indicative of higher parasympathetic activity, which is considered more adaptive. Among other functions, parasympathetic activity of the autonomic nervous system elicits a state of relaxation, resting, or calm. Higher HRV is associated with younger age, better physical fitness, and better overall health, while lower levels of HRV have been linked to depression, anxiety, negative affect, high stress, and burnout [55–57]. A meta-analysis of research to date has supported the robust utility of HRV as a measure of stress [56].

Connectivity issues prevented HRV readings from being recorded for 8 participants, resulting in HRV readings for 93 participants. The reading for each participant was automatically cleaned to eliminate artifact by the Elite HRV software using algorithms they have developed for this purpose [58, 59]. LnRMSSD and SDNN scores were then calculated for each person. We examined the data points for irregularities and dropped one data point that was irregular. Average reading time for the 92 respondents was 5.03 min (SD = 1.13, range = 2.15 to 7.46).

##### Hair cortisol

Cortisol is a downstream hormone secreted by the adrenal glands when the HPA axis is stimulated. It can be measured in a variety of specimens, including blood, saliva, urine, and hair [60]. Cortisol is produced primarily in hair follicles and incorporated into the hair as it grows. Levels within a specific hair segment reflect cumulative cortisol secretion within that hair growth period [61, 62]. Conceptually, the accumulation of high levels of cortisol over time may provide an indication of chronic or sustained stress over time, with each 1 cm of hair from the scalp assessing stress levels for the prior month [6, 7]. There are no specified cut-offs for cortisol levels and stress, but, on average, cortisol levels are higher in people with chronic stress as well as those with various health conditions [60]. One reference range reported for cortisol in hair is 17.7–153.2 pg/mg of hair (median 46.1 pg/mg) [63]. A more recent study [64] reported a hair cortisol concentration reference interval in healthy individuals with low levels of stress to be 40–128 pg/mg of hair while the range for concentrations in stressed individuals was higher (182–520 pg/mg of hair). Hair specimens in our sample were obtained from 44 respondents, mostly because respondents did not have enough hair to provide a sample. Only one person with enough hair refused to provide a sample. Samples were sent to the Stress Physiology Investigative Team (SPIT) lab at Iowa State University for analysis (details of their analytic process are in Additional file 2). Values for two respondents, with very high cortisol concentrations (i.e. > 235.23 pg/mg) were winsorized (transforming extreme values to minimize the influence of outliers) to fall within 3 standard deviations [8].

### Independent variables

The survey with providers included questions regarding individual level and situational factors that have been theorized or found to be associated with stress and burnout in prior studies [35, 65]. The facility survey with the maternity head acquired information about situational factors at the facility such as staffing and availability of resources level. All variables below were obtained from the individual surveys, except those indicated as facility level (see study questionnaire in additional file 1 for how all variables were assessed).

***Individual factors:***

- *Demographic factors*: age, gender, marital status, parity
- *Socioeconomic factors*: education, income, perceived social status, and perceived accomplishments (described in Table 1)
- *Physical Health*: self-rated health status, chronic disease, and exercise

**Table 1:** Provider perception questions from survey

**Perceived social status question stem**: During the survey, research assistants were instructed to show respondents a drawing of a ladder with 10 rungs and read this stem to them:	
*“This ladder represents where people stand in Kenya. At the top of the ladder are the people who are the best off, those who have the most money, most education, and best jobs. At the bottom are the people who are the worst off, those who have the least money, least education, worst jobs, or no job.”*	
After reading the question stem, they then read the following questions:	
*“Thinking of when you were growing up (before you had your own family and before you became a health care provider), where will you place your family’s social status on this ladder?” and “Thinking of now, where will you place your social status? Select the rung that best represents where you think you stand now on the ladder?”*	
The selected ranks were used to measure ***Perceived social status of family growing up*** and ***Perceived social status now*** respectively.	
**Perceived accomplishments:** This was measured by asking the question: “*Thinking of what you wish you will have accomplished at this stage in your life, would you say you have accomplished less than you hoped, exactly what you hoped, or more than you hoped?”*	
**Perceived availability of work supplies**: This was measured by asking the question: *“On a scale of 0 to 10, where 0 means that you don’t have any of the things you need to effectively do your work, such as medicines and supplies, and 10 means you have everything you need to work with, where will you place your situation in this facility?”*	
**Effort-reward imbalance and overcommitment**: These were both measured with the Effort Reward Imbalance Questionnaire: a validated 16 item measure based on the work stress model to assess the balance between efforts spent (3 items), rewards received (7 items), and commitments (6 items) (Siegrist, Li, & Montano, 2014). Each item has responses on a 4-point scale from strongly disagree to strongly agree (see additional file 1 for questions).	
***Effort Reward Imbalance*** was calculated as the effort score (the sum of the 3 effort items) divided by reward score (the sum of the 7 reward items) multiplied by a correction factor (k = 7/3) used to adjust for the unequal number of items of the effort and reward scores. Higher scores indicate more effort reward imbalance.	
***Overcommitment*** was calculated as the sum of responses to 6 commitment items. Scores range from 6 to 24, with higher scores indicating higher commitment to one’s work.	

***Situational factors related to job demands and resources:***

- *Contextual factor:* facility type (facility level)
- *Role and experience*: position and years of experience
- *Workload:* overcommitment (described in Table 1). Number of workdays per week and work hours per day. Number of providers (doctors, clinical officers, nurses, and auxiliary staff) at the facility and number usually on duty during the day and at night; and average monthly deliveries (facility level).
- *Availability of resources*: perceived availability of work supplies. Availability of essential commodities (based on a composite score of combined responses regarding availability of blood, IV infusions, uterotonics, MgSO4, and general supplies); caesarian section capability; and consistency of water and electricity (facility level).
- *Experience of traumatic events*: personal experience with maternal and neonatal patient deaths. Number of maternal deaths, stillbirths, and neonatal deaths recorded in the facility in the last year (facility level).
- *Stressful interpersonal interactions*: perceived disrespect from supervisors, colleagues, or patients
- *Effort-reward imbalance*: balance between efforts spent and rewards received (see Table 1)

### Analysis

Data were imported into STATA and merged for quantitative analysis. Preliminary analysis involved factor analysis of the perceived stress and burnout items to assess construct validity, and Cronbach’s alpha to assess internal consistency reliability. We performed these assessments to assure their appropriateness for our sample before generating the summative scores.

We used descriptive statistics (means and proportions) to examine the distribution of the dependent and independent variables. We then examined the bivariate associations between the variables using crosstabulations, correlations, and unadjusted linear regressions. The scores for the psychological measures were approximately normally distributed, so untransformed scores are used for the bivariate and multivariate analysis. For HRV, we used the lnRMSSD which corrects for positive skewness. Cortisol levels were also positively skewed, which was corrected with a log transformation.

Because a number of providers were selected from each facility, we considered multilevel models to account for the clustering. However, the intraclass correlations for the null models were generally low (0.12 for perceived stress, 0.05 for burnout, 0.01 for HRV), except for cortisol which was 0.32. *P*-values for the Likelihood Ratio tests comparing multilevel vs. single level linear models were not significant at 0.05 or less for any of the outcome measures, suggesting the multilevel model was not significantly different from the ordinary least squares (OLS) model [66]. However, because of differences by facility that emerged in other analyses, we employed a conservative approach, computing multilevel models in the final analyses with facility as level 2. We used restricted maximum likelihood (REML) because of its tendency for less bias in small samples [67]. We also ran the final models as OLS models with robust standard errors to account for clustering within facilities in sensitivity analysis. Only variables that were significant in bivariate models for at least one of the outcome measures were included in the multivariate models. Models were then tested for model fit and collinearity and we removed variables that did not improve the model or were strongly correlated.

Finally, using structural equation modeling, we tested if the relationship between significant situational factors (i.e. overcommitment) and burnout was mediated by perceived stress, after accounting for the other individual and situational factors. The indirect (mediated) effect was the difference between the coefficient for the specific predictor in the burnout model without the mediator (total effect: c) and the coefficient in the model that included the mediator (direct effect: c’). The percentage mediated by perceived stress was calculated as the indirect effect divided by the total effect ((c-c’)/c) times 100 [68, 69]. We did not test the mediated effect of perceived stress on the physiological measures because they were not correlated in bivariate models. We used STATA 15.0 for all analyses [70].

## Results

### Descriptive results

#### Demographics

Of the 101 providers who participated, there were 62 nurses/midwives, 15 clinical officers, 1 doctor, and 23 other staff grouped together as support staff (7 ward aids and 14 cleaners; 1 technician and 1 pharmacist). Forty-two worked in government hospitals, 45 in government health centers and dispensaries, and 14 in private or mission facilities. Sixty-three were female and 81 were less than 40 years old. About half had been health care providers for 5 years or less. Because of missing data on the outcome measures, the analytic sample is 87 for perceived stress, 97 for burnout, 92 for HRV and 44 for cortisol; the distribution of respondents is relatively similar in each of these analytic samples (Table 2).

**Table 2:** Participant demographics and selected independent variables

Analytic sample	***Full Sample (N = 101)***		***Perceived stress (N = 87)***		***Burnout (N = 97)***		***HRV (N = 92)***		***Hair cortisol (N = 44)***	
	No.	%	No.	%	No.	%	No.	%	No.	%
***Demographic factors***										
Gender										
Male	38	37.6	33	37.9	38	39.2	34	37.0	1	2.3
Female	63	62.4	54	62.1	59	60.8	58	63.0	43	97.7
Age										
23 to 29 years	32	31.7	26	29.9	32	33	30	32.6	14	31.8
30 to 39	49	48.5	42	48.3	46	47.4	43	46.7	19	43.2
40 to 52 years	20	19.8	19	21.8	19	19.6	19	20.7	11	25.0
Marital status										
Married	75	74.3	64	73.6	72	74.2	67	72.8	35	79.5
All Single	26	25.7	23	26.4	25	25.8	25	27.2	9	20.5
Number of children a										
No children	22	22.0	18	20.9	21	21.9	22	24.2	7	16.3
1 to 3	56	56.0	48	55.8	54	56.2	48	52.7	24	55.8
4 to 7	22	22.0	20	23.3	21	21.9	21	23.1	12	27.9
***Socioeconomic factors***										
Education level										
Less than College	18	17.8	15	17.2	16	16.5	18	19.6	11	25.0
College and above	83	82.2	72	82.8	81	83.5	74	80.4	33	75.0
Monthly salary a										
Less than 10,000 KSh	20	20.2	16	18.8	18	18.9	20	22.2	10	23.3
10,000 to less than 50,000 KSh	40	40.4	34	40.0	40	42.1	35	38.9	17	39.5
50,000 KSh or more	39	39.4	35	41.2	37	38.9	35	38.9	16	37.2
Perceived social status of family growing up										
Bottom half	85	84.2	74	85.1	83	85.6	80	87.0	36	81.8
Upper half	16	15.8	13	14.9	14	14.4	12	13.0	8	18.2
Perceived social status of self now										
Bottom half	57	56.4	48	55.2	55	56.7	54	58.7	26	59.1
Upper half	44	43.6	39	44.8	42	43.3	38	41.3	18	40.9
Perceived accomplishments in life a										
Less than you hoped	84	84.0	72	83.7	81	84.4	75	82.4	38	88.4
Exactly what you hoped	13	13.0	11	12.8	13	13.5	13	14.3	4	9.3
More than you hoped	3	3.0	3	3.5	2	2.1	3	3.3	1	2.3
***Physical Health***										
Self-rated health										
Fair/Poor	21	20.8	19	21.8	20	20.6	20	21.7	10	22.7
Good/Very good/Excellent	80	79.2	68	78.2	77	79.4	72	78.3	34	77.3
Has chronic health condition	14	13.9	13	14.9	14	14.4	13	14.1	7	15.9
Frequency of exercise a										
Never/less than once a week	60	60.0	53	61.6	57	59.4	53	58.2	30	69.8
Once or more per week	40	40.0	33	38.4	39	40.6	38	41.8	13	30.2
***Contextual factors***										
Facility type										
Govt. Hospital	42	41.6	36	41.4	39	40.2	42	45.7	20	45.5
Govt. Health Center/Dispensary	45	44.6	40	46.0	44	45.4	36	39.1	16	36.4
Mission/Private Hospital	14	13.9	11	12.6	14	14.4	14	15.2	8	18.2
***Position and experience***										
Position										
Nurse/Midwife	62	61.4	54	62.1	61	62.9	54	58.7	28	63.6
Clinical officer/Doctor	16	15.8	13	14.9	15	15.5	15	16.3	3	6.8
Support staff	23	22.8	20	23.0	21	21.6	23	25.0	13	29.5
Years as provider										
0 to 5 years	50	49.5	39	44.8	48	49.5	49	53.3	21	47.7
6 to 10 years	38	37.6	37	42.5	37	38.1	31	33.7	20	45.5
More than 10 years	13	12.9	11	12.6	12	12.4	12	13.0	3	6.8
***Workload***										
Workdays per week										
5 or fewer days	90	89.1	79	90.8	86	88.7	81	88.0	40	90.9
More than 5 days	11	10.9	8	9.2	11	11.3	11	12.0	4	9.1
Work hours per day										
8 or fewer hours	60	59.4	50	57.5	58	59.8	56	60.9	28	63.6
More than 8 h	41	40.6	37	42.5	39	40.2	36	39.1	16	36.4
***Availability of resources***										
Perceived availability of work tools										
Half or less of the time	64	63.4	56	64.4	61	62.9	59	64.1	30	68.2
More than half the time	37	36.6	31	35.6	36	37.1	33	35.9	14	31.8
***Traumatic experiences***										
Ever experienced maternal/neonatal death	42	41.6	36	41.4	40	41.2	39	42.4	17	38.6
Maternal/neonatal death in last year	24	57.1	20	55.6	23	57.5	21	53.8	10	58.8
***Stressful interpersonal interactions***										
Experienced disrespect from superior in last year	34	34.3	29	34.1	33	34.7	31	34.4	17	40.5
Experienced disrespect from colleague in last year	38	37.6	33	37.9	37	38.1	35	38.0	15	34.1
Experienced disrespect from patient in last year	56	55.4	48	55.2	53	54.6	51	55.4	27	61.4
***Effort reward imbalance***										
Below median ERI score	39	39.4	31	36.0	37	38.9	36	40.0	19	45.2
Above median ERI score	60	60.6	55	64.0	58	61.1	54	60.0	23	54.8
***Overcommitment score:*** mean (SD)	100	16.7 (2.18)	86	16.8 (2.20)	96	16.7 (2.19)	91	16.7 (2.20)	43	16.7 (2.24)

#### Stress and burnout levels

The average PSS score from the 10 items was 20.7 (SD = 4.3) (Table 3). Based on recommended cut offs, participant scores indicate that 85% had moderate stress and 11.5% had high stress. The average burnout score was 3.0 (SD = 0.9). Based on the specified cut offs, 65% would be classified as having low burnout and 19.6% as high burnout. The HRV score (RMSSD) was 60.5 (SD = 33.0) and mean cortisol was mean cortisol was 44.2 pg/mg (SD = 60.9)). Perceived stress and burnout were correlated (r = 0.34, *p* < 0.001), but HRV and hair cortisol levels were not significantly correlated with each other nor with perceived stress or burnout. Although not reaching a level of significance, the correlation between cortisol and perceived stress (r = 0.26) suggest that higher cortisol level might be associated with greater perceived stress if increased power was available from a larger sample size.

**Table 3:** Distribution of outcome measures

Continuous stress variables	N	Mean	SD	Median	Min	Max
Perceived stress score (0 to 40)	87	20.7	4.30	21.0	11.0	32.0
Burnout score (1-7)	97	3.0	0.90	3.1	1.0	5.1
Physical fatigue	97	3.4	1.03	3.7	1.0	6.0
Emotional exhaustion	97	2.5	1.27	2.3	1.0	5.7
Cognitive weariness	97	2.8	1.08	3.0	1.0	4.8
HRV measures						
Rmssd	92	60.5	33.0	54.2	15.6	167.6
lnRmssd	92	4.0	0.54	4.0	2.7	5.1
Sdnn	92	75.6	50.05	62.5	21.6	287.2
Hair cortisol level (pg/mg)	44	44.2	60.88	20.5	3.8	236.2
log Cortisol	44	3.2	1.03	3.0	1.3	5.5
**Categorical stress variables**	**N**	**%**				
Perceived stress from PSS						
Low stress (0–13)	3	3.5				
Moderate stress (14–26)	74	85.1				
High stress (27–40)	10	11.5				
Burnout from SMBS						
No burnout (≤2.0)	15	15.5				
Low burnout (2.1 to 3.75)	63	65.0				
High burnout (≥3.75)	19	19.6				

#### Individual and situational predictors

Average days of work per week was 5.1 (89% work 5 or fewer days per week) with 8.7 h per day (59% work 8 h or less per day). Average perceived availability of work supplies was 5.1 out of 10 (63% had work tools and supplies only half or less than half the time). 58% had experienced the death of a mother or baby during pregnancy or childbirth at some time in their career, with 43% of those occurring in the last year. About a third experienced disrespect from a superior or colleague in the last year, and over half experienced disrespect from a patient (Table 2). For the facility level variables, 74% worked in a facility with no doctor and 25% in a facility with no clinical officers. Most (81%) worked in a facility where there were usually only one or two clinical providers on duty during the day and only 1 or no provider on duty at night (62%).

### Bivariate analysis

In bivariate regressions, higher education, income, perceived social status, and perceived accomplishments were significantly associated with lower perceived stress, while overcommitment was associated with higher stress (see Additional file 3 for crosstabulations and coefficients from bivariate linear regressions). Higher education and income were also associated with lower burnout, while being female as well as having higher overcommitment and effort reward imbalance were associated with higher burnout. For the physiological measures, those younger than 30 years, single, and nulliparous had higher HRV scores than those over 30 years, married, and with children respectively. For cortisol, only provider’s perceived social status was significant, with slightly lower cortisol among those who perceived themselves to be in the upper half of the social status. None of the other individual level variables were significantly associated with any of outcome measures. Bivariate associations for facility level variables are also shown in Additional file 3. However, these variables were correlated with each other and with the type of facility rather than with the study outcomes. Thus, they were not included in the final multivariate models. Instead, we included facility type as a predictor in the multivariate models, in addition to facility as the cluster variable in multilevel models.

### Multivariate analysis

#### Perceived stress

In our final multilevel multivariate analysis, only providers’ perceived accomplishments and overcommitment to work were associated with perceived stress (Table 4). Those who felt they had accomplished what they hoped had lower perceived stress scores than those who felt they have achieved less than they hoped (β= − 2.83; CI = -5.47; − 0.18); and each unit increase in overcommitment to work was associated with a 0.6 higher perceived stress score (CI: 0.19, 1.03).

**Table 4:** Multilevel multivariate linear regression on outcome measures

	*Perceived stress*			*Burnout*			*HRV (lnRmssd)*			*Cortisol (log)*		
	*β*	*[95% CI]*		*β*	*[95% CI]*		*β*	*[95% CI]*		*β*	*[95% CI]*	
Facility type												
Govt. Hospital												
Govt. Health Center/Dispensary	−0.004	[−2.10	2.10]	−1.06	[−7.78	5.67]	0.17	[− 0.14	0.48]	0.47	[− 0.55	1.49]
Mission/Private Hospital	−0.21	[−3.67	3.25]	−4.23	[− 14.4	5.89]	−0.078	[− 0.55	0.39]	− 0.12	[− 1.74	1.51]
Position												
Nurse/Midwife												
Clinical officer/Doctor	−0.054	[−2.94	2.84]	−5.42	[−12.9	2.11]	−0.029	[−0.38	0.32]	−0.69	[−2.38	1.00]
Support staff	0.69	[−3.47	4.85]	2.42	[−8.07	12.9]	0.49*	[0.011	0.97]	0.11	[−1.36	1.59]
Gender												
Male												
Female	0.88	[−1.20	2.96]	5.17+	[−0.29	10.6]	0.039	[− 0.22	0.30]	0.92	[−1.43	3.27]
Age												
23 to 29 years												
30 to 39	0.53	[−1.96	3.03]	−1.06	[−7.61	5.49]	− 0.47*	[− 0.78	− 0.16]	− 0.09	[−1.20	1.02]
40 to 52 years	− 0.41	[− 3.33	2.52]	−0.79	[−8.63	7.05]	− 0.49*	[− 0.85	− 0.13]	−0.24	[− 1.39	0.90]
Marital status												
Married												
All Single	−1.06	[−3.45	1.33]	4.4	[−1.69	10.5]	0.25+	[−0.020	0.53]	0.27	[−0.81	1.36]
Monthly salary a												
Less than 10,000 KSh												
10,000 to < 50,000 KSh	0.28	[−3.82	4.39]	−2.85	[−13.4	7.64]	0.36	[−0.12	0.83]	0.29	[−1.37	1.95]
50,000 KSh or more	−0.94	[−5.42	3.55]	1.05	[−9.88	12.0]	0.45+	[−0.040	0.95]	0.28	[−1.62	2.19]
Work hours per day												
8 or fewer hours												
More than 8 h	1.15	[−0.32	2.63]	−1.02	[−4.97	2.94]	−0.098	[−0.29	0.093]	−0.06	[− 0.70	0.58]
Perceived accomplishments in life a												
Less than hoped												
Exactly/more than hoped	−2.83*	[−5.47	−0.18]	0.74	[−6.18	7.67]	−0.033	[−0.34	0.28]	−0.34	[−1.66	0.99]
Overcommitment score	0.61*	[0.19	1.03]	2.05**	[0.91	3.19]	0.0033	[−0.049	0.056]	0.031	[−0.15	0.21]
Constant	9.84*	[1.27	18.4]	7.36	[−15.3	30.0]	3.69***	[2.66	4.72]	1.54	[−2.96	6.04]
*Random effects*												
Health facility: Identity sd (_cons)	0.00	0.00	0.00	4.67	1.95	11.17	0.21	0.04	0.96	0.55	0.12	2.61
Sd (Residual)	4.00	3.38	4.72	10.48	8.65	12.69	0.47	0.37	0.61	0.96	0.60	1.53
icc (health facility)	0.00	0.00	0.00	0.17	0.03	0.58	0.16	0.01	0.85	0.25	0.01	0.94
No. of groups	30			30			28			24		
No. of observations	83			93			88			41		

95% confidence intervals in brackets. + *p* < 0.10; * p < 0.05; ** *p* < 0.01; *** *p* < 0.001

#### Burnout

Only 2 variables emerged as significant or trending toward significant in the final model for burnout (Table 5). Increasing overcommitment was associated with higher burnout (β=2.05, CI = 0.91, 3.19); and females had a 5-point higher burnout score on average than males (*p* = 0.06).

**Table 5:** Structural equation model for mediation of perceived stress on burnout (*N* = 79)

	***Burnout***								
	*Total effects*			*Direct effects*			*Indirect effects*		
	*β*	*[95% CI]*		*β*	*[95% CI]*		*β*	*[95% CI]*	
*Perceived stress scores*	0.69*	0.08	1.29	0.69*	0.08	1.29			
Overcommitment score	2.03***	0.91	3.15	1.63*	0.49	2.77	0.4+	−0.05	0.84
Perceived accomplishments in life a									
Less than hoped									
Exactly/more than hoped	−1.33	−8.39	5.73	0.74	−6.35	7.82	−2.07	−4.57	0.43
Facility type									
Govt. Hospital									
Govt. Health Center/Dispensary	−3.06	−8.75	2.63	−3.27	−8.79	2.25	0.21	−1.19	1.61
Mission/Private Hospital	−4.78	−13.88	4.32	−4.78	−13.6	4.05	−0.01	−2.22	2.21
Position									
Nurse/Midwife									
Clinical officer/Doctor	−3.36	−11.08	4.37	−3.08	− 10.57	4.42	−0.28	−2.18	1.62
Support staff	−1.52	−12.43	9.38	−2	−12.58	8.59	0.47	−2.22	3.16
Gender									
Male									
Female	6.44*	0.81	12.07	6.05*	0.58	11.52	0.39	−1.03	1.8
Age									
23 to 29 years									
30 to 39	1.73	−4.87	8.32	1.5	−4.9	7.89	0.23	−1.39	1.85
40 to 52 years	−0.53	−8.43	7.37	−0.11	−7.77	7.56	−0.42	−2.38	1.54
Marital status									
Married									
All Single	3.34	−3.05	9.73	4.21	−2.03	10.46	−0.88	−2.62	0.86
Monthly salary a									
Less than 10,000 KSh									
10,000 to < 50,000 KSh	−8.75	−19.66	2.16	−8.76	−19.34	1.82	0.01	−2.65	2.67
50,000 KSh or more	−6.98	−18.94	4.98	−6.13	−17.75	5.49	−0.85	−3.86	2.16
Work hours per day									
8 or fewer hours									
More than 8 h	−1.53	−5.47	2.4	−2.21	−6.07	1.65	0.68	−0.45	1.8

95% confidence intervals in brackets. + *p* < 0.10; * *p* < 0.05; ** *p* < 0.01; *** *p* < 0.001

#### Heart rate variability

A number of individual level variables were significantly associated with HRV (see Table 4). Support staff had a higher lnRMSSD than nurses and clinical officers, while older providers had a lower lnRMSSD than younger providers (*p* < 0.05). Also, single providers and those with higher income had higher lnRMSSD than married providers and those with lower income respectively (*p* < 0.1).

***Cortisol.*** None of the individual or situational variables were significantly associated with cortisol in the final multivariate analysis—potentially due to the much smaller analytic sample. However, the direction of associations for most variables was in general consistent with that obtained for the other measures, with slightly higher cortisol levels among providers in health centers, support staff, females, and single providers and among those who felt less accomplished and overcommitted.

#### Mediation analysis

The results from the structural equation model are shown in Table 5. It shows that higher perceived stress was associated with greater burnout (β=0.69; CI: 0.08, 1.29). Perceived stress accounted for 19.6% (p=0.08) of the effect of overcommitment on burnout, suggesting perceived stress partially mediates the effect of overcommitment on burnout.

#### Sensitivity analysis

The results from additional analyses using OLS regression and sub-samples were in general consistent with the main results in the direction and magnitude of associations.

## Discussion

This cross-sectional study of maternity health providers in a rural county revealed a high level of stress and burnout. Nearly all the providers (96%) had moderate to high levels of stress and more than 8 out of 10 had some level of burnout, with 20% having high levels of burnout that represent cause for clinical concern. A perceived sense of accomplishment in life emerged as a protective factor to stress whilst excessive overcommitment to one’s work was predictive for both high perceived stress and burnout among these providers. In addition, female providers had higher burnout scores compared to male providers. Perceived stress partially mediated the effect of overcommitment on burnout. Support staff, single providers, and those with higher income had higher HRV than clinical providers, married providers, and those with lower income respectively, while older providers had a lower HRV than younger providers. Although the association between cortisol levels and all the predictors were not statistically significant, the effect sizes and direction of associations in the final model indicated a potential relationship with perceived stress as well as with predictors such as gender, provider role, accomplishment in life, and overcommitment to work if sample size was larger and the power to detect significant effects was greater.

The high levels of perceived stress and burnout are consistent with similar studies with maternal health providers (although not directly comparable because of the use of the different measures) [28, 71–73]. For example, Muriithi and Kariuki found that 88.6% of the nurses working in a maternity hospital in Kenya were experiencing burnout measured with the Maslach Burnout Inventory-Human Services Survey (MBI-HSS) [29]. Another study among Ugandan midwives in two rural districts also reported a burnout rate of 88% based on the Professional Quality of Life Scale [27]. The burnout rates from our study are also consistent with results from other studies among healthcare workers in Kenya. Kokonya et al. reported a burnout rate of 95% among providers at a national hospital in Kenya based on the Compassion Fatigue Self-Test [74]. In another study among providers at a psychiatric hospital in Kenya, 87% reported low to high emotional exhaustion and 87% reported depersonalization, measured by the MBI-HSS [75]. These high rates of burnout are potentially due to a generally stressful work environment for healthcare providers, which has been recognized as a crisis globally [76]. In addition, maternity providers tend to carry a heavy workload burden with high demands on their time and physical performance, while balancing professional standards and expectations from childbearing women and their families [24, 73]. They are also exposed to higher levels of trauma from traumatic birth experiences, which could increase burnout among them [27, 77]. Given the negative effects of high stress and burnout on job performance [78, 79], and individual physical and emotional well-being [80], the findings underscore the need for interventions with both structural and individual targets.

We did not find studies on perceived stress among health care workers in Kenya, but the levels of perceived stress from our study are similar to a study in the same county that recorded a mean PSS score of 19 (SD = 4) among pregnant women [46]. Studies with health care workers elsewhere have also reported high perceived stress levels [81–84]. One study among nurses in a hospital in the United states reported that 92% of nurses had moderate-to-very high stress levels [81] which is similar to the 96% we found. We identified no studies on HRV or cortisol levels among health care workers in Africa with which to compare our results.

Our findings related to perceived accomplishments and overcommitment are of great interest and are consistent with theories of stress and burnout [50, 85]. The association we found between providers’ satisfaction with their accomplishment in life and less self-reported stress may be because a sense of personal accomplishment appears to moderate the effect of work demands on perceived stress [86, 87]. Conversely, a sense that one’s accomplishments are minimal may suggest decreased professional efficacy which is a manifestation of burnout [7].

We found that overcommitment predicted both higher stress and burnout. Overcommitment to work, called ‘workaholism’ in some research, has been related previously to higher job stress and burnout [88] and is associated with two particular dimensions of job burnout: emotional exhaustion and depersonalization [89]. Overcommitment has also been related to a concept called ‘drive,’ reflecting internal pressure to work and frequent thoughts about work [90]. It has been proposed that individuals with high work drive may stretch themselves in many directions in the hope of being able to handle situations on the job. While they may appear to cope well with challenges at work, they often perceive their own attempts to cope as ineffective. As a result, they may experience ‘incomplete recovery from mentally and physically demanding tasks’—particularly if they experience low control and low anticipation of rewards from the work [91]. This lack of recovery would likely lead to high stress and burnout over time. Overcommitment to one’s work has also been linked to role ambiguity and increased task demands—both factors that are predictive of stress and burnout in various studies [78, 92, 93]. Role ambiguity may be especially high among support staff who are often called upon to undertake duties for which they may have not received adequate training due to the heavy patient load —including assisting births. There were, however, no significant differences in the outcome measures by position type, except for the higher HRV levels among support staff, which suggests support staff may have a more adaptive response. These factors need further research in this context to better understand their relationships.

The finding that female maternal health providers had higher levels of burnout compared to their male counterparts is consistent with findings from other studies, although a meta-analysis showed that this relationship is not consistently identified [94]. Abraham et al. showed that goal directed coping tendencies related to occupational satisfaction and well-being were higher in males compared to female providers [95]. This may offer an explanation to the gender differences. Another plausible explanation, considering the context of a rural setting like Migori Kenya, would be that strict gender roles still persist and the redistribution of roles at home to match increased role responsibilities outside the home is still lacking. Consequently, female providers have a greater workload as they attempt to balance both home and work responsibilities. Of note, the non-significant associations between other demographic factors and perceived stress and burnout have been reported in other studies [87].

No previous study in Africa has reported on the correlation between biological measures of stress and socio-demographic factors among healthcare workers. As noted earlier, greater HRV is generally considered more adaptive since it reflects the ability of the ANS to dynamically adjust to changes in the environment. Our finding that lower HRV is associated with older age is consistent with the fact that the parasympathetic response decreases with age [96, 97]. This has implications for older providers’ ability to physiologically adapt in efficient ways to stressors they encounter at work. Experience may however influence the effect on perceived stress. We also found lower HRV among those with lower income. This may indicate that financial strain takes a toll on the cardiovascular system over time, reducing its adaptive capacity. Financial strain is a source of stress that may, in turn, affect HRV. Although not identified in our results, previous research has shown stress to be associated with lower HRV [55–57]. The average cortisol level obtained in our study is significantly higher than that from a prior study with pregnant women receiving antenatal care in the same county (44.2 ± 60.88 compared 6.11 ± 1.04 pg/mg) [46]. This difference in cortisol levels indicates a more heightened state of arousal for providers than for maternity patients, a state in which they are mobilized and ready for action. Still, based on ranges found in previous studies, providers in our study did not appear to have excessively high cortisol levels. The fact that no individual or situational factors appeared to predict providers’ cortisol levels was likely the result of the smaller sample size we had for these analyses. Given the association between high cortisol levels, stress, and adverse health outcomes [60, 98], research with a larger sample size is essential to understand potential factors that may place providers at risk for dysregulation of the HPA axis.

A key strength of this study is the use of both psychological and physiological measures of stress. As expected, there was a positive correlation between perceived stress and burnout, with stress partially mediating the effect of overcommitment on burnout. But there were no significant correlations between the other measures. This is not surprising since psychological and physiological measures assess different components of stress with different underlying mechanisms. Prior studies have also shown an inconsistent relationship between psychological and physiologic measures of stress (e.g. [99, 100]). In addition, our physiological measures assessed different stress response systems and periods of time, with HRV being an acute five-minute measure of current ANS response and hair cortisol being a retrospective, longer term measure of HPA axis response. Thus, the likelihood of HRV and cortisol being related is reduced. Further, we don’t know whether these HRV levels reflected their tonic or general state over the past months or were primarily in response to the research-specific situation.

Our study is limited by the small sample size in a rural population. In particular, the lack of significant association between hair cortisol and the predictors may be due to the much lower sample size for the hair cortisol analysis. However, the findings are validated by the fact that in general, the direction of associations are consistent with that from the psychosocial measures. In addition, the high self-reported stress and burnout in the sample reduced the variation in responses, which might have contributed to the lack of significant associations with most predictors. Future studies with a larger sample in more diverse settings are thus needed to allow for generalization of findings. Despite these limitations, we recruited various cadres of staff providing maternal health care, whereby previous studies have been focused only on midwives (nursing cadre). Subsequently, we found comparable levels of stress among support staff in maternity care, emphasizing the need for interventions for all cadres providing maternity care. Lastly, to our knowledge, this is the first study that examined both psychological and physiological measures of stress among healthcare workers in Kenya and sub-Saharan Africa. We have shown that these measures can be reliably applied to this population and demonstrated the high stress and burnout experienced by maternity providers.

## Conclusions

In this exploratory study of stress and burnout among maternity providers in a rural county in Kenya, we found many providers experienced high levels of stress and burnout. Both individual and work-related factors contributed to this high stress and burnout. Given the effects of stress and burnout on provider wellbeing, quality of care, and the efficiency of the healthcare workforce, it is important that interventions are designed to help providers manage stress and prevent burnout. Interventions are needed to prevent the stressors where possible, or to help them develop positive coping mechanisms to respond to the stressors. Assisting providers in reducing their overcommitment and identifying its causes are particularly essential in light of the significant role played by overcommitment in both stress and burnout. Helping providers identify and value their personal life accomplishments is also important in decreasing stress. In addition, interventions are needed for those already experiencing burnout to prevent adverse effects on the health of individual providers as well as the health system. This should include strengthening psychosocial support systems for health care workers. Future studies should also seek to more fully understand the sources of stress in this population, examine providers’ perceptions of the stressors, and coping mechanisms they employ to inform appropriate interventions.

## Supplementary Information

**Additional file 1.** Study questionnaire. Portions of study questionnaire with questions used in this manuscript including perceived stress and burnout measures.

**Additional file 2.** Hair cortisol analysis procedures. Details of procedure for hair cortisol analysis.

**Additional file 3.** Additional results. Results of bivariate analysis.

## Data Availability

The data supporting the conclusions of this article is included within the article and its additional files. Additional data available from lead author upon reasonable request

## Footnotes

1 https://elitehrv.com/
