# Supplementary material for: Psychological and physiological stress and burnout among maternity providers in a rural county in Kenya: individual and situational predictors
Source: BMC Public Health. 2021 Mar 6;21:453. doi: 10.1186/s12889-021-10453-0 (PMC7936594; doi:10.1186/s12889-021-10453-0)
Supplement: Supplementary file 1 — Additional file 1. Study questionnaire. Portions of study questionnaire with questions used in this manuscript including perceived stress and burnout measures. [file 12889_2021_10453_MOESM1_ESM.pdf]

PROVIDER INFORMATION

12 How long have you worked as a health provider? ______________________

ENTER NUMBER IN YEARS
ENTER 0 IF LESS THAN 1 YEAR

ENTER 99 IF THEY REFUSE TO ANSWER

13 How long have you worked in this facility? ______________________

ENTER NUMBER IN YEARS
ENTER 0 IF LESS THAN 1 YEAR

ENTER 99 IF THEY REFUSE TO ANSWER

14 What is your current position in this facility?

- ◯ 0 Doctor (Medical officer)
- ◯ 1 Doctor (Specialist/Consultant)
- ◯ 2 Clinical officer
- ◯ 3 Nurse
- ◯ 4 Midwife
- ◯ 5 Ward aid/assistant
- ◯ 6 Cleaner
- ◯ 7 Cook
- ◯ 8 Lab Technician
- ◯ 9 Blood Bank worker
- ◯ 10 Pharmacist
- ◯ 11 Other (specify)________
- ◯ 99 Refused to answer

14b if other specify ______________________

15 (Don't ask this just select based on response above)

- ◯ 0 No
- ◯ 1 Yes

Is the respondent a clinician-i.e doctor, clinical officer, nurse/midwife?

16 How long have you been in this position? ______________________

ENTER NUMBER IN YEARS
ENTER 0 IF LESS THAN 1 YEAR

ENTER 99 IF THEY REFUSE TO ANSWER

17 About how many days do you currently work per week?

- ◯ 1
- ◯ 2
- ◯ 3
- ◯ 4
- ◯ 5
- ◯ 6
- ◯ 7
- ◯ 99 Refused to answer

18 About how many hours do you currently work per day?

ENTER APPROXIMATE NUMBER OF HOURS BETWEEN 0 AND 24 HOURS

ENTER 99 IF THEY REFUSE TO ANSWER

____

19 In general, how satisfied are you with your job? Will you say you are very satisfied, satisfied, dissatisfied, or very dissatisfied?

- ◯ 0 Very dissatisfied
- ◯ 1 Dissatisfied
- ◯ 2 Neither satisfied nor dissatisfied
- ◯ 3 Satisfied
- ◯ 4 Very satisfied
- ◯ 99 Refused to answer

EFFORT-REWARD IMBALANCE SCALE

The following items refer to how you feel about your present job. For each of the following statements, please indicate whether you strongly agree, agree, disagree or strongly disagree.

20	ERI1 I have constant time pressure due to a heavy work load.	◯ 0 Strongly disagree<br>◯ 1 Disagree<br>◯ 2 Agree<br>◯ 3 Strongly agree<br>◯ 99 Refused to answer
21	ERI2 I have many interruptions and disturbances while performing my job	◯ 0 Strongly disagree<br>◯ 1 Disagree<br>◯ 2 Agree<br>◯ 3 Strongly agree<br>◯ 99 Refused to answer
22	ERI3 Over the past few years, my job has become more and more demanding.	◯ 0 Strongly disagree<br>◯ 1 Disagree<br>◯ 2 Agree<br>◯ 3 Strongly agree<br>◯ 99 Refused to answer
23	ERI4 I receive the respect I deserve from my superior or some one to whom I report.	◯ 0 Strongly disagree<br>◯ 1 Disagree<br>◯ 2 Agree<br>◯ 3 Strongly agree<br>◯ 99 Refused to answer
24	ERI5 My job promotion prospects are poor.	◯ 0 Strongly disagree<br>◯ 1 Disagree<br>◯ 2 Agree<br>◯ 3 Strongly agree<br>◯ 99 Refused to answer
25	ERI6 I have experienced or I expect to experience an undesirable change in my work situation.	◯ 0 Strongly disagree<br>◯ 1 Disagree<br>◯ 2 Agree<br>◯ 3 Strongly agree<br>◯ 99 Refused to answer
26	ERI7 My job security is poor.	◯ 0 Strongly disagree<br>◯ 1 Disagree<br>◯ 2 Agree<br>◯ 3 Strongly agree<br>◯ 99 Refused to answer
27	ERI8 Considering all my efforts and achievements, I receive the respect and prestige I deserve at work.	◯ 0 Strongly disagree<br>◯ 1 Disagree<br>◯ 2 Agree<br>◯ 3 Strongly agree<br>◯ 99 Refused to answer
28	ERI9 Considering all my efforts and achievements, my job promotion prospects are adequate.	◯ 0 Strongly disagree<br>◯ 1 Disagree<br>◯ 2 Agree<br>◯ 3 Strongly agree<br>◯ 99 Refused to answer

29 ERI10 Considering all my efforts and achievements, my salary / income is adequate.

- ◯ 0 Strongly disagree
- ◯ 1 Disagree
- ◯ 2 Agree
- ◯ 3 Strongly agree
- ◯ 99 Refused to answer

30 OC1 I get easily overwhelmed by time pressures at work.

- ◯ 0 Strongly disagree
- ◯ 1 Disagree
- ◯ 2 Agree
- ◯ 3 Strongly agree
- ◯ 99 Refused to answer

31 OC2 As soon as I get up in the morning I start thinking about work problems

- ◯ 0 Strongly disagree
- ◯ 1 Disagree
- ◯ 2 Agree
- ◯ 3 Strongly agree
- ◯ 99 Refused to answer

32 OC3 When I get home, I can easily relax and 'switch off' work.

- ◯ 0 Strongly disagree
- ◯ 1 Disagree
- ◯ 2 Agree
- ◯ 3 Strongly agree
- ◯ 99 Refused to answer

33 OC4 People close to me say I sacrifice too much for my job.

- ◯ 0 Strongly disagree
- ◯ 1 Disagree
- ◯ 2 Agree
- ◯ 3 Strongly agree
- ◯ 99 Refused to answer

34 OC5 Work rarely lets me go, it is still on my mind when I go to bed.

- ◯ 0 Strongly disagree
- ◯ 1 Disagree
- ◯ 2 Agree
- ◯ 3 Strongly agree
- ◯ 99 Refused to answer

35 OC6 If I postpone something that I was supposed to do today I'll have trouble sleeping at night.

- ◯ 0 Strongly disagree
- ◯ 1 Disagree
- ◯ 2 Agree
- ◯ 3 Strongly agree
- ◯ 99 Refused to answer

COHEN STRESS SCALE

The following questions ask about your feelings and thoughts during THE PAST MONTH.

In each question, you will be asked HOW OFTEN you felt or thought a certain way. Although some of the questions are similar, there are small differences between them and you should treat each one as a separate question. The best approach is to answer fairly quickly. That is, don t try to count up the exact number of times you felt a particular way, but tell me the answer that in general seems the best.

For each statement, please tell me if you have had these thoughts or feelings: never, almost never, sometimes, fairly often, or very often.

(Read all answer choices each time)

36 B.1. In the past month, how often have you been upset because of something that happened unexpectedly?

- ◯ 0 Never
- ◯ 1 Almost Never
- ◯ 2 Sometimes
- ◯ 3 Fairly Often
- ◯ 4 Very Often
- ◯ 99 Refused to answer

37 B.2. In the past month, how often have you felt unable to control the important things in your life?

- ◯ 0 Never
- ◯ 1 Almost Never
- ◯ 2 Sometimes
- ◯ 3 Fairly Often
- ◯ 4 Very Often
- ◯ 99 Refused to answer

38 B.3. In the past month, how often have you felt nervous or stressed?

- ◯ 0 Never
- ◯ 1 Almost Never
- ◯ 2 Sometimes
- ◯ 3 Fairly Often
- ◯ 4 Very Often
- ◯ 99 Refused to answer

39 B.4. In the past month, how often have you felt confident about your ability to handle personal problems?

- ◯ 0 Never
- ◯ 1 Almost Never
- ◯ 2 Sometimes
- ◯ 3 Fairly Often
- ◯ 4 Very Often
- ◯ 99 Refused to answer

40 B.5. In the past month, how often have you felt that things were going your way?

- ◯ 0 Never
- ◯ 1 Almost Never
- ◯ 2 Sometimes
- ◯ 3 Fairly Often
- ◯ 4 Very Often
- ◯ 99 Refused to answer

41	B.6. In the past month, how often have you found that you could not cope with all the things you had to do?	◯ 0 Never<br>◯ 1 Almost Never<br>◯ 2 Sometimes<br>◯ 3 Fairly Often<br>◯ 4 Very Often<br>◯ 99 Refused to answer
42	B.7. In the past month, how often have you been able to control irritations in your life?	◯ 0 Never<br>◯ 1 Almost Never<br>◯ 2 Sometimes<br>◯ 3 Fairly Often<br>◯ 4 Very Often<br>◯ 99 Refused to answer
43	B.8. In the past month, how often have you felt that you were on top of things?	◯ 0 Never<br>◯ 1 Almost Never<br>◯ 2 Sometimes<br>◯ 3 Fairly Often<br>◯ 4 Very Often<br>◯ 99 Refused to answer
44	B.9. In the past month, how often have you been angry because of things that happened that were outside of your control?	◯ 0 Never<br>◯ 1 Almost Never<br>◯ 2 Sometimes<br>◯ 3 Fairly Often<br>◯ 4 Very Often<br>◯ 99 Refused to answer
45	B.10. In the past month, how often have you felt that difficulties were piling up so high that you could not overcome them?	◯ 0 Never<br>◯ 1 Almost Never<br>◯ 2 Sometimes<br>◯ 3 Fairly Often<br>◯ 4 Very Often<br>◯ 99 Refused to answer

BURNOUT MEASURE

Next I am going to ask you some questions on how you feel at Work.

Below are a number of statements that describe different feelings that you may feel at work. Please indicate how often, in the past 30 workdays, you have felt each of the following feelings:

Please tell me if it is:

0, Never or almost never |
2, Very infrequently |
3, Quite infrequently |
4, Sometimes |
5, Quite frequently |
6, Very frequently |
7, Always or almost always

In the past 30days how often have you felt this way at work?

46	P1. I feel tired	◯ 0 Never or almost never<br>◯ 1 Very infrequently<br>◯ 2 Quite infrequently<br>◯ 3 Sometimes<br>◯ 4 Quite frequently<br>◯ 5 Very frequently<br>◯ 6 Always or almost always<br>◯ 99 Refused to answer
47	P2. I have no energy for going to work in the morning	◯ 0 Never or almost never<br>◯ 1 Very infrequently<br>◯ 2 Quite infrequently<br>◯ 3 Sometimes<br>◯ 4 Quite frequently<br>◯ 5 Very frequently<br>◯ 6 Always or almost always<br>◯ 99 Refused to answer
48	P3. I feel physically drained	◯ 0 Never or almost never<br>◯ 1 Very infrequently<br>◯ 2 Quite infrequently<br>◯ 3 Sometimes<br>◯ 4 Quite frequently<br>◯ 5 Very frequently<br>◯ 6 Always or almost always<br>◯ 99 Refused to answer

49 P4. I feel fed up
- 0 Never or almost never
- 1 Very infrequently
- 2 Quite infrequently
- 3 Sometimes
- 4 Quite frequently
- 5 Very frequently
- 6 Always or almost always
- 99 Refused to answer

50 P5. I feel like my "batteries" are "dead"
- 0 Never or almost never
- 1 Very infrequently
- 2 Quite infrequently
- 3 Sometimes
- 4 Quite frequently
- 5 Very frequently
- 6 Always or almost always
- 99 Refused to answer

51 P6. I feel burned out
- 0 Never or almost never
- 1 Very infrequently
- 2 Quite infrequently
- 3 Sometimes
- 4 Quite frequently
- 5 Very frequently
- 6 Always or almost always
- 99 Refused to answer

52 C7. My thinking process is slow
- 0 Never or almost never
- 1 Very infrequently
- 2 Quite infrequently
- 3 Sometimes
- 4 Quite frequently
- 5 Very frequently
- 6 Always or almost always
- 99 Refused to answer

53 C8. I have difficulty concentrating
- 0 Never or almost never
- 1 Very infrequently
- 2 Quite infrequently
- 3 Sometimes
- 4 Quite frequently
- 5 Very frequently
- 6 Always or almost always
- 99 Refused to answer

54 C9. I feel I'm not thinking clearly
- 0 Never or almost never
- 1 Very infrequently
- 2 Quite infrequently
- 3 Sometimes
- 4 Quite frequently
- 5 Very frequently
- 6 Always or almost always
- 99 Refused to answer

55 C10. I feel I'm not focused in my thinking

- 0 Never or almost never
- 1 Very infrequently
- 2 Quite infrequently
- 3 Sometimes
- 4 Quite frequently
- 5 Very frequently
- 6 Always or almost always
- 99 Refused to answer

56 C11. I have difficulty thinking about complex things

- 0 Never or almost never
- 1 Very infrequently
- 2 Quite infrequently
- 3 Sometimes
- 4 Quite frequently
- 5 Very frequently
- 6 Always or almost always
- 99 Refused to answer

57 E12. I feel I am unable to be sensitive to the needs of coworkers and patients

- 0 Never or almost never
- 1 Very infrequently
- 2 Quite infrequently
- 3 Sometimes
- 4 Quite frequently
- 5 Very frequently
- 6 Always or almost always
- 99 Refused to answer

58 E13. I feel I am not capable of investing emotionally in coworkers and patients

- 0 Never or almost never
- 1 Very infrequently
- 2 Quite infrequently
- 3 Sometimes
- 4 Quite frequently
- 5 Very frequently
- 6 Always or almost always
- 99 Refused to answer

59 E14. I feel I am not capable of being sympathetic to co-workers and patients

- 0 Never or almost never
- 1 Very infrequently
- 2 Quite infrequently
- 3 Sometimes
- 4 Quite frequently
- 5 Very frequently
- 6 Always or almost always
- 99 Refused to answer

Heart rate variability measurement

60 Now I am going to put this devise on your finger while we continue with the rest of the questions. The device measures your heart rate variability, which can be used to assess your level of stress.

Please remain seated and still while we take the reading as movement can affect the results. We will take the reading for 5 mins.

Agreed to HRV testing?

- ◯ 0 No
- ◯ 1 Yes
- ◯ 2 Sensor not available
- ◯ 3 Sensor not connecting

61 Time sensor put on respondent

____

67	On a scale of 0 to 10, where 0 means that you don't have any of the things you need to effectively do your work, such as medicines and supplies, and 10 means you have everything you need to work with, where will you place your situation in this facility?	◯ 0<br>◯ 1<br>◯ 2<br>◯ 3<br>◯ 4<br>◯ 5<br>◯ 6<br>◯ 7<br>◯ 8<br>◯ 9<br>◯ 10<br>◯ 99 Refused to answer
68	Within the last year, have you been treated by a superior in a way that was disrespectful or humiliating?<br>(If they say yes, ask)<br>How often will you say this has occurred?	◯ 0 No, never<br>◯ 1 Yes, a few times<br>◯ 2 Yes, many times<br>◯ 3 Yes, always<br>◯ 99 Refused to answer
69	Within the last year, have you been treated by a colleague at work in a way that was disrespectful or humiliating?<br>(If they say yes, ask)<br>How often will you say this has occurred?	◯ 0 No, never<br>◯ 1 Yes, a few times<br>◯ 2 Yes, many times<br>◯ 3 Yes, always<br>◯ 99 Refused to answer
70	Within the last year, have you been treated by a patient in a way that was disrespectful or humiliating?<br>(If they say yes, ask)<br>How often will you say this has occurred?	◯ 0 No, never<br>◯ 1 Yes, a few times<br>◯ 2 Yes, many times<br>◯ 3 Yes, always<br>◯ 99 Refused to answer
71	Have you ever lost a mother or baby during pregnancy or childbirth?<br><br>That is a pregnant woman you were involved in caring for died, her baby was delivered stillbirth, or the mother or baby died soon after delivery?	◯ 0 No<br>◯ 1 Yes<br>◯ 2 Prefer not to answer
72	(If yes): I am sorry to hear that: Was this a maternal death, still birth, or early neonatal death?	◯ 0 Maternal death<br>◯ 1 Still birth<br>◯ 2 Neonatal death<br>◯ 3 Other death<br>◯ 99 Refused to answer
73	Did this happen in the last year?	◯ 0 No<br>◯ 1 Yes<br>◯ 2 Prefer not to answer
74	Have you ever had any thoughts of suicide, i.e. wanting to end you life?	◯ 0 No<br>◯ 1 Yes<br>◯ 2 Prefer not to answer
75	Have you ever attempted suicide?	◯ 0 No<br>◯ 1 Yes<br>◯ 2 Prefer not to answer

Health

Next, I am going to ask some general questions about your health and lifestyle

93 How will you rate your health now? Will you say it is excellent, very good, good, fair, poor, or very poor?

- ◯ 0 Very Poor
- ◯ 1 Poor
- ◯ 2 Fair
- ◯ 3 Good
- ◯ 4 Very Good
- ◯ 5 Excellent
- ◯ 99 Refused to answer

94 How often do you do moderate to vigorous exercise (such as running, fast walking, or going to the gym) ?

- ◯ 0 Almost never
- ◯ 1 Less than once a week
- ◯ 2 About 1 to 2 days a week
- ◯ 3 About 3 to 5 days a week
- ◯ 4 Almost everyday
- ◯ 99 Refused to answer

95 How frequently do you drink alcohol

- ◯ 0 Almost never
- ◯ 1 Less than once a week
- ◯ 2 About 1 to 2 days a week
- ◯ 3 About 3 to 5 days a week
- ◯ 4 Almost everyday
- ◯ 99 Refused to answer

96 Do you smoke?

- ◯ 0 No
- ◯ 1 Yes
- ◯ 99 Refused to answer

97 Do you use any substances like cocaine, marijuana or other hard drugs?

- ◯ 0 No
- ◯ 1 Yes
- ◯ 99 Refused to answer

98 What substance do you use?

____

99 About how many hours do you sleep each night?

- ◯ 0 Less than 6 hours
- ◯ 1 About 6 to 8 hours
- ◯ 2 About 9 to 10 hours
- ◯ 3 More than 10 hours
- ◯ 99 Refused to answer

100 Do you have any chronic health condition like hypertension, diabetes, HIV, arthritis, depression, etc.?

- ◯ 0 No
- ◯ 1 Yes
- ◯ 99 Refused to answer

100 If yes: What chronic health condition do you have? (select all that apply)

- ☐ 1 Hypertension
- ☐ 2 Diabetes
- ☐ 3 HIV
- ☐ 4 Arthritis
- ☐ 5 Depression
- ☐ 8 Asthma
- ☐ 9 Other
- ☐ 99 Refused to answer

100bif other specify

____

101 Are you currently taking any medications for this condition?

◯ 0 No
◯ 1 Yes
◯ 99 Refused to answer

102 What type of medications are you taking? (select all that apply)

☐ 1 Pain killer
☐ 2 Hypertension drugs
☐ 3 Diabetes drugs
☐ 4 HIV drugs
☐ 5 Anti-allergic
☐ 6 Anti-depressant
☐ 7 Antimalarial
☐ 8 Antibiotic
☐ 9 Sleep medicine
☐ 10 Asthma medicine
☐ 11 Other
☐ 99 Refused to answer

102bif other specify

____

103 Have you taken any medicine today?

◯ 0 No
◯ 1 Yes
◯ 99 Refused to answer

104 What medicine did you take

☐ 1 Pain killer
☐ 2 Hypertension drugs
☐ 3 Diabetes drugs
☐ 4 HIV drugs
☐ 5 Anti-allergic
☐ 6 Anti-depressant
☐ 7 Antimalarial
☐ 8 Antibiotic
☐ 9 Sleep medicine
☐ 10 Asthma medicine
☐ 11 Other
☐ 99 Refused to answer

104bif other specify

____

105 Was this medicine prescribed by a health care provider?

◯ 0 No
◯ 1 Yes
◯ 99 Refused to answer

DEMOGRAPHICS:

I will end by asking you a few last questions about your self.

166 Enter Sex of provider
- ◯ 0 Male
- ◯ 1 Female

167 How old are you ?
(enter age in years)

enter 999 if they don't know

____

168 What is your current marital status?
- ◯ 1 Single
- ◯ 2 Partnered/Cohabiting
- ◯ 3 Married
- ◯ 4 Widowed
- ◯ 5 Divorced/Separated
- ◯ 99 Refused to answer

169 How many children do you have

____

170 What is the highest level of school you attended?
- ◯ 0 No school
- ◯ 1 Attended primary but did not finish
- ◯ 2 Primary
- ◯ 3 Post-primary/vocational
- ◯ 4 Secondary
- ◯ 5 College(middle level)
- ◯ 6 University or above
- ◯ 99 Refused to answer

171 Do you come from Migori County?
- ◯ 0 No
- ◯ 1 Yes
- ◯ 99 Refused to answer

172 How long have you lived in this county?

ENTER NUMBER IN YEARS
ENTER 0 IF LESS THAN 1 YEAR

____

173 What is your religion?
- ◯ 0 Catholic
- ◯ 1 Methodist/Presby/Anglican
- ◯ 2 Protestant/Pentecostal
- ◯ 3 Seventh Day Adventist
- ◯ 4 Other Christian
- ◯ 5 Muslim
- ◯ 6 Other religion
- ◯ 7 No religion
- ◯ 99 Refused to answer

174 if other specify

____

175 (SHOW RESPONDENTS A DRAWING OF A LADDER WITH 10 RUNGS, AND READ THIS THEM)

This ladder (SHOW SHEET WITH LADDER) represents where people stand in Kenya.
At the top of the ladder are the people who are the best off, those who have the most money, most education, and best jobs. At the bottom are the people who are the worst off, those who have the least money, least education, worst jobs, or no job.

Thinking of when you were growing up (before you had your own family and before you became a health care provider), where will you place your family's social status on this ladder.
Select the rung that best represents where you think your family stood on the ladder.

- ◯ 1
- ◯ 2
- ◯ 3
- ◯ 4
- ◯ 5
- ◯ 6
- ◯ 7
- ◯ 8
- ◯ 9
- ◯ 10
- ◯ 99 Refused to answer

---

176 Thinking of now, where will you place your social status?
Select the rung that best represents where you think you stand now on the ladder.

- ◯ 1
- ◯ 2
- ◯ 3
- ◯ 4
- ◯ 5
- ◯ 6
- ◯ 7
- ◯ 8
- ◯ 9
- ◯ 10
- ◯ 99 Refused to answer

---

177 About how much do you earn in a month from this position (i.e. your net income)?
(amount in KSH)
ENTER 999 IF THEY REFUSE

____

This is the end of our interview. Thank you so much for taking the time to answer our questions.

197

Do you have any questions for me about this interview? __________________________________

198 TIME INTERVIEW ENDED ____________________________

199 For study personnel: Did you enter data online in REDCAP during interview?

- ◯ 0 Yes, Data entered in REDcap during interview
- ◯ 1 No, Data entered on hard copy and entered into RED cap later

200

(Write any comments about this interview you will want to make here) __________________________________
