# Supplementary material for: Psychological and physiological stress and burnout among maternity providers in a rural county in Kenya: individual and situational predictors
Source: BMC Public Health. 2021 Mar 6;21:453. doi: 10.1186/s12889-021-10453-0 (PMC7936594; doi:10.1186/s12889-021-10453-0)
Supplement: Supplementary file 2 — Additional file 2. Hair cortisol analysis procedures. Details of procedure for hair cortisol analysis. [file 12889_2021_10453_MOESM2_ESM.pdf]

Hair sample analysis process

Each sample was segmented by 3cm (N= 33) if they had enough length, otherwise, their original length was kept, between 2cm-2.9cm (N = 11). Segmented samples were then washed twice in 5ml of high-performance liquid chromatography (HPLC) isopropanol with constant rotation. Hair was dried under forced air for 3-4 hours in a fume hood to dry. Dried samples were weighted between 5 and 15 mg depending on their amounts. Weighed samples were ground in a ball mill for 8 minutes at 30hz. The powdered hair was then extracted in 1.5ml of methanol over a 24-hour incubation period using continuous inversion at room temperature. After the incubation period, samples were centrifuged at 5000rpm for 5 minutes before 1ml of supernatant was transferred to a new clean vial and dried under a nitrogen evaporator in a water bath (50 °C). The extract was then reconstituted with 150µL of kit-provided assay diluent and assayed using commercially available enzyme-immunoassay kits (Salimetrics, PA). The Inter-assay C.V. (between plates) was 1.70 and Intra-assay C.V. (between samples) was 5.54. The cortisol levels for each respondent were examined for irregularities.
